# Case Report: A Child With Hemophilia A Serves as Donor for Hematopoietic Stem Cell Transplantation to Cure His Brother’s Severe Aplastic Anemia

**DOI:** 10.3389/pore.2022.1610171

**Published:** 2022-06-08

**Authors:** Gabriella Kertész, Krisztián Kállay, Csaba Kassa, Marianna Zombori, Imre Bodó, Csongor Kiss, István Szegedi, Gergely Kriván

**Affiliations:** ^1^ Department of Pediatric Hematology and Stem Cell Transplantation, Central Hospital of Southern Pest, National Institute of Hematology and Infectious Diseases, Budapest, Hungary; ^2^ Department of Infectious Diseases, Semmelweis University, Budapest, Hungary; ^3^ Kemény Pál Pediatric Onco-Hematologic Department, Heim Pál National Pediatric Institute, Budapest, Hungary; ^4^ Department of Internal Medicine and Haematology, Semmelweis University, Budapest, Hungary; ^5^ Division of Pediatric Hematology-Oncology, Department of Pediatrics, Faculty of Medicine, University of Debrecen, Debrecen, Hungary

**Keywords:** hemophilia a, severe aplastic anemia (SAA), hematopoietic stem cell transplantation (HSCT), hepatitis associated bone marrow failure (HABMF), children

## Abstract

The first-line treatment of severe aplastic anemia is allogeneic hematopoietic stem cell transplantation with a matched sibling donor. However, co-morbidities of the identical donor can make donation difficult. We present a transplantation where in parallel with the patient’s conditioning treatment, the preparation of the donor with severe hemophilia A required a special management with perioperative factor VIII substitution. Donation was successful without complications, and 18 months after transplantation, the patient and his donor are well without any long-term sequelae. To our knowledge, this is the first reported succesfull transplantation with hemophilic child serving as a bone marrow donor. The procedure did not mean a significant risk to donor health, so donors with hemophilia should not be excluded from donation.

## Highlights

• What is the new aspect of your work? Evaluating hemophilic patient as feasible donor for stem cell transplantation
• What is the central finding of your work? Hematopoietic stem cell transplantation can be performed safely and does not mean unacceptable risk to hemophilic donor
• What is (or could be) the specific clinical relevance of your work?This is the first case in the literature and can help other groups to consider hemophilic donor feasibility and not exclude them from donation

## Introduction

Acquired severe aplastic anemia (SAA) is a rare and potentially fatal disease. It is characterized by pancytopenia and a hypocellular bone marrow. The treatment of choice in childhood is hematopoietic stem cell transplantation (HSCT) with a matched sibling donor. Severe hemophilia A is an inherited bleeding disorder due to factor VIII (FVIII) deficiency. Patients suffer from serious bleeding episodes; however, they can live with a normal quality of life on proper prophylactic FVIII substitution.

There are documented cases of patients with hemophilia who were successfully treated by solid organ transplantation, mostly liver transplant performed for different indications (1). However, there are only few data about solid organ donation from living donors with hemophilia. Furthermore, there are no data on hematopoietic stem cell donation in donors with severe hemophilia A. Presumably, this is because of the associated risks of excessive bleeding; concern about inhibitor development after a period of intensive factor replacement; and a perceived risk of transmission of hemophilia or pathogens (e.g., hepatotropic viruses) from the donor to the recipient.

## Case Presentation

We present here an 11-year-old male patient with SAA, who underwent HSCT with his human leukocyte antigen (HLA)-identical sibling donor. The patient presented with sudden onset jaundice and was diagnosed with acute hepatitis (GPT: 2411 U/L, GOT: 1728 U/L, GGT: 129 U/L, LDH: 556U/l, AP: 406 U/L, bilirubin: 152 µmol/L, conjugated bilirubin: 138 μmol/L, WBC: 3.33 G/L, ANC: 1.66 G/L, platelets: 45 G/L, Hgb: 133 g/L, no coagulation abnormality). No viral infection was found with serology and polymerase chain reactions, autoimmune diseases were ruled out by autoantibody tests and iron and copper metabolism investigations did not detect metabolic diseases. Platelet transfusions were needed to correct progressive thrombocytopenia. A bone marrow aspirate was inadequate to assess cellularity but ruled out a malignant hematologic disorder.

High-dose glucocorticoid treatment was initiated due to the hypothesis of immune-mediated hepatitis-associated immune thrombocytopenia (ITP). The treatment remained ineffective, and severe three-lineage pancytopenia evolved. He was transferred to a tertiary care center where liver and bone marrow biopsies were performed. Liver histology with aspecific findings supported the etiology of viral or toxic origin. Bone marrow histology showed a cellularity of only 5 percent, the remaining cells were mostly lymphocytes and plasmocytes. Erythropoesis with more than 15 erytroblasts were only found in 3 marrow spaces, and the erythropoesis was normoblastic. Megakaryoblasts and granolocyte precursors were not found. Activated T-lymphocytes were found in the bone marrow by flow cytometry. SAA was diagnosed based on our findings. The clinical course was consistent with hepatitis associated bone marrow failure (HABMF). The patient was transferred to the stem cell transplantation unit for further evaluation and treatment of bone marrow failure (WBC: 0.24 G/L, ANC: 0.04 G/L, hemoglobin: 87 g/L, platelet: 13 G/L). A second confirmatory trephine biopsy was performed 2 weeks later as recommended by the EWOG-SAA protocol and we found 5% cellularity and only lymphocytes and plasma cells in the bone marrow; so, the diagnosis of hepatitis-associated SAA was confirmed.

The search for a family donor identified a 13-years old HLA-identical brother, who has been treated for severe hemophilia A. He was diagnosed because of bleeding after blood sampling in the perinatal period with a factor VIII level below 1%. There were no genetic investigation available at the time of diagnosis. Since there is no one in the family with bleeding disorders or symptoms the patient probably harvest a new mutation. He received on demand factor prophylaxis for the first 3 years of life, then was switched to regular prophylaxis (PPX) by recombinant FVIII preparations. The patient had no spontaneous bleedings on PPX, extra factor replacement was only required a few times due to minor traumatic bleeding events. Thus, the brother had more than 1000 exposure days (EDs) to FVIII products, without prior inhibitor development. After discussion with the primary physicians, other hemostasis and transplantation experts and the family, this brother was chosen as a suitable donor for the bone marrow transplantation.

**FIGURE 1:**
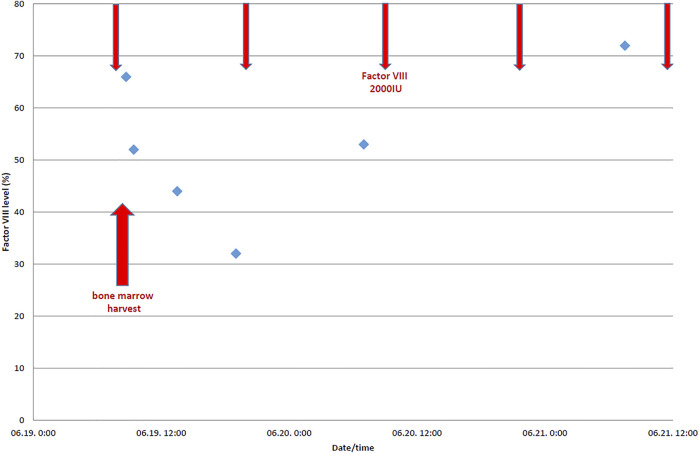
Perioperative factor VIII levels (%) of the donor with severe hemophilia A.

Following recommendations of international guidelines we concluded that the required perioperative factor level of the donor in case of a low-risk procedure should be not less than 30%–50% (2). FVIII was planned to be given in bolus, two times per day, and duration of the intensive substitution was planned for 2 postoperative days. The preoperative factor replacement (2000 IU moroctocog alpha) was administered 30–60 min before the procedure. The next dose was scheduled 12 h after the procedure, or earlier in case of bleeding or a suboptimal FVIII level, monitored during and after the procedure. The level of factor VIII was measured pre-, intra- and postoperatively to ascertain that the target level of >30% is met (as shown in Table 1 and Figure 1). No periprocedural bleeding was observed. The donor was discharged on the 3rd day after the procedure without any complications (2, 3).

**TABLE 1:** Factor VIII levels (%).

Date/Time	FVIII level (%)	Remarks
2019. 6. 7. 11:48	7	On admission
2019. 6. 17. 10:41	3	during preparation
2019. 6. 19. 8:42	66	Preoperative
2019. 6. 19. 9:25	52	Intraoperative
2019. 6. 19. 13:30	44	Postoperative
2019. 6. 19. 19:00	32	Late night after procedure
2019. 6. 20. 7:00	53	Next morning
2019. 6. 21. 7:30	72	Third morning
2019. 6. 24. 10:00	29	Three days later

The HSCT was performed 1 month after the first and 2 weeks after the second bone marrow biopsy; so within a month after diagnosis (better outcome expected with shorter time frame). The following conditioning therapy was used: fludarabine: 30 mg/m^2^/day for 4 days, cyclophosphamide: 25 mg/kg/day for 4 days, and Thymoglobulin (Sanofi Genzyme Corporation, Cambridge, Massachusetts, United States), 2.5 mg/kg/day for 3 days. The patient received 4.46 x 10^6^/kg CD34^+^ stem cells from a bone marrow graft (BM). Graft versus host disease (GVHD) prophylaxis consisted of cyslosporin-A (started 1 day before transplantation, then modified according to the serum level monitored 2 times weekly) and short-course methotrexate (10 mg/m^2^ on day 1, 3, and 6). The transplant course was complicated by a febrile neutropenia caused by *Streptococcus mitis,* however, this bloodstream infection did not progress to cardiovascular deterioration and was controlled by targeted antibiotics. On day +8 he complained of right eye and face pain, a swollen zygomatic region was palpable. Traditional X-ray and otolaryngologist diagnosed acute inflammation of the right sinus maxillaris, the serum galactomannan level elevated. We considered this entity a probable invasive fungal infection and intravenous liposomal amphotericin-B therapy was initiated. The CT examination performed later revealed no bone destruction in the region or any sign of fungal infection in the lungs. After a neutrophil engraftment achieved on the day 21 the patient was discharged on D40 with full donor chimerism (96%) detected by short tandem repeat method and complete hematological reconstitution, requiring no hemosubstitution. Immune-reconstitution was detected on day +63 with CD4^+^ T-cell number of 207/µl. Donor chimerism dropped to 88% at day +96 but it is usual with reduced intensity conditioning and we observed no sign of graft failure.

A clinically insignificant adenovirus reactivation was observed with low DNA copy numbers requiring no antiviral therapy but frequent monitoring. Therefore, the central venous catheter was removed just 5 months after transplantation. A prolonged activated partial thromboplastin time (aPTT) was detected by the perioperative hemostasis assessment. The prolonged aPTT could not be corrected by 1:1 mixture of normal plasma, and a lupus anticoagulant (LA) was detected by phospholipid neutralization which remained positive during follow-up without other antiphospholipid antibodies. No causal relationship was found with either the transplantation or the donor’s hemophilia A, FVIII levels were normal. There were no bleeding or thrombotic complications. We did not find the cause of LA but it has no impact on the recipient’s well-being. The immunosuppression therapy was stopped 8 months after transplantation. Almost 3 years after transplantation the recipient has normal blood counts, the patient continues to attend school, revaccination protocol is completed and no long-term toxicity is present. His FVIII level remains normal. The donor is also well on day +1036 after bone marrow harvest. Inhibitor production was not detected throughout the entire observation period.

## Discussion

In this presentation we highlight all the ethical and medical considerations which lead us to choose a patient with severe hemophilia A as a hematopoietic stem cell donor for his younger brother, who was diagnosed with severe aplastic anemia. To our knowledge, this is the first description of hematopoietic stem cell donation by a person with severe hemophilia A. The protection of donor’s health is of utmost importance in the hematopoietic stem cell transplant setting. Even minimal risk expected to the donor are only acceptable in case of outstanding advantages to the recipient’s survival.

We had to consider three major aspects of accepting a patient with hemophilia as a hematopoietic stem cell donor. The patient suffered from a potentially lethal disease for what sibling HSCT represented a curative procedure with excellent results. Major concerns regarding donor health included risk of perioperative bleeding (bone marrow harvest) and risk of inhibitor development due to the high-dose factor substitution in the perioperative setting. Last but not least we wanted to exclude the possibility of any disease-transmission to the recipient.

These issues were widely discussed with hematologists and experts in hemophilia and stem cell transplantation. Randomized controlled studies about the optimal intensity and duration of perioperative factor substitution therapy in the pediatric population are rare. The routine care of children is based mostly on data and experiences from adult patients and the pilot pediatric perioperative trials of the factor preparations during regulatory approval (4). Since the introduction of early FVIII prophylaxis in children with hemophilia, orthopedic surgery for intra-articular bleeding has been rarely required. Common surgical interventions in pediatric patients with hemophilia include: tonsillectomy, circumcision, dental procedures and insertion of a central venous devices (4). Whereas no data were found in the literature on bone marrow harvest in patients with hemophilia, first we had to estimate the risk of perioperative bleeding. This procedure was declared as a low-risk intervention due to the facts that 1) the insertion wound is small (aspiration needle); 2) the posterior superior iliac spine is an excellent surface for compression (in case of bleeding), and 3) the bone marrow is very rich in tissue factor.

Our second concern has been the risk of inhibitor development due to the extra factor substitution during a procedural setting. Several trials have shown that an early and high intensity substitution therapy may be associated with the risk of inhibitor development (5). However, most of the inhibitors occurred early in the introductory phase of substitution (i.e., 90 percent occurred within the first 16 exposure days). No antibody producing potential was ever reported due to a short-term substitution required for minor surgical interventions. Our observation provides further evidence that such risk is minimal or non-existent (6, 7). We discussed the potential benefits of administering short-term immune-suppression therapy to the donor to prevent inhibitor development, but decided against it because of the former observations and potential infections.

Although hemophilia is a hematological disease, factor production is not related to the hematopoietic system. For a long time, it has been widely accepted that the main site of FVIII production is the liver. It was based upon several observations that hemophilia resolved after liver transplantation carried out for other reasons (1, 8). Recent studies with knockout mice, however, identified the endothelial cells as the primary source of FVIII production (9–11). The rich endothelial lining of the liver may explain the former observation. Thus, the transmission of hemophilia from the stem cell donor with hemophilia to the recipient with SAA was not expected by bone marrow transplantation. Indeed, the recipient’s FVIII levels remained normal throughout the observation period. The transmission of hepatotropic viruses was a major concern in the era of blood-derived factor replacement products. Since the donor received only recombinant FVIII concentrates, such a risk was virtually non-existent, and, indeed, the patient was negative for all hepatitis and HIV serology.

Moreover, the proper graft source was discussed in this case. Generally, bone marrow graft is recommended in aplastic anemia because of the lower probability of graft versus host disease in this non-malignant disease, where the graft versus leukemia effect is not necessary. Additionally, we had to discuss it in the aspect of donor safety issues in this case with the hemophilic donor. There is a slightly higher chance of local bleeding during harvesting a bone marrow (BM) graft than collecting a peripheral stem cell graft (PBSC) with apheresis. However, splenic enlargement and rupture is more frequent during cytokine mobilization and extracorporeal anticoagulation in the PBSC setting. We considered the bleeding from the harvesting site a much more easily locally controllable complication than intra-abdominal issues, so we decided beside the BM graft.

Due to these risk factors, alternative therapies to cure the patient were considered. However, both immunosuppressive therapy (with antithymocyte globulin) and first line unrelated HSCT offered significantly inferior survival to our patient, while the recommended first line therapy of sibling bone marrow transplantation promised over 90 percent event free survival. We considered the preceding hepatitis as an additional burden and therefore chose the least hepatotoxic reduced intensity conditioning possible. We hypothesized that the liver disease is of the same origin, probably immune mediated, as the aplastic anemia. Therefore we were not so much concerned about transplant related liver injuries, such as veno-occlusive disease (VOD), but we administered prophylactic heparin and ursodeoxycholic acid. Indeed, the hepatitis resolved with the administering of the conditioning regimen. The dilemma of the parents’ informed consent was addressed: the decision to consent the procedure of their both children was supported by the Institutional Ethical Committee.

## Conclusion

To our knowledge we present here the first case of hematopoietic stem cell donation from a donor with hemophilia. The bone marrow harvest was carried out with specially prepared and controlled high-dose perioperative FVIII substitution without complications. The patient with severe aplastic anemia engrafted on time without serious complications. One and a half years later, both children are alive and well without any sequel. Our experience suggests that a person with hemophilia A could be considered as a suitable related donor for stem cell transplantation. The procedure does not mean harm for donor or patient safety so donors with hemophilia should not be excluded from donation. Hemophilia A is not transmittable by stem cell transplantation.

## Data Availability

The original contributions presented in the study are included in the article/supplementary material, further inquiries can be directed to the corresponding author.

## Abbreviations

BM, bone marrow; EWOG-SAA, European Working Group on Severe Aplastic Anemia in Children; FVIII, factor VIII; GVHD, graft versus host disease; HABMF, hepatitis associated bone marrow failure; HSCT, hematopoietic stem cell transplantation; ITP, immune thrombocytopenia; LA, lupus anticoagulant; PBSC, peripheral blood stem cell; PPX, prophylaxis; SAA, severe aplastic anemia.
